# Supplementary figures and images for: Involvement of enhanced expression of classical complement C1q in atherosclerosis progression and plaque instability: C1q as an indicator of clinical outcome
Source: PLoS One. 2022 Jan 27;17(1):e0262413. doi: 10.1371/journal.pone.0262413 (PMC8794146; doi:10.1371/journal.pone.0262413)

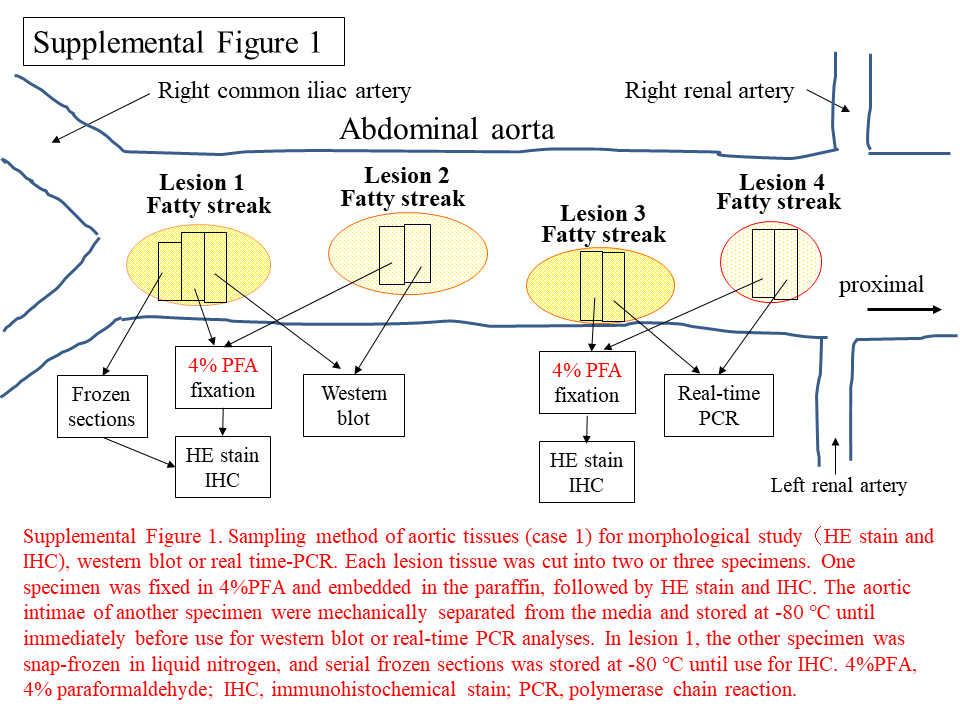

Supplement: S1 Fig — Each lesion tissue was cut into two or three specimens. One specimen was fixed in 4%PFA and embedded in the paraffin, followed by HE stain and IHC. The aortic intimae of another specimen were mechanically separated from the media and stored at -80°C until immediately before use for western blot or real-time PCR analyses. In lesion 1, the other specimen was snap-frozen in liquid nitrogen, and serial frozen sections was stored at -80°C until use for IHC. 4%PFA, 4% paraformaldehyde; IHC, immunohistochemical stain; PCR, polymerase chain reaction. (TIF) [file pone.0262413.s001.TIF]

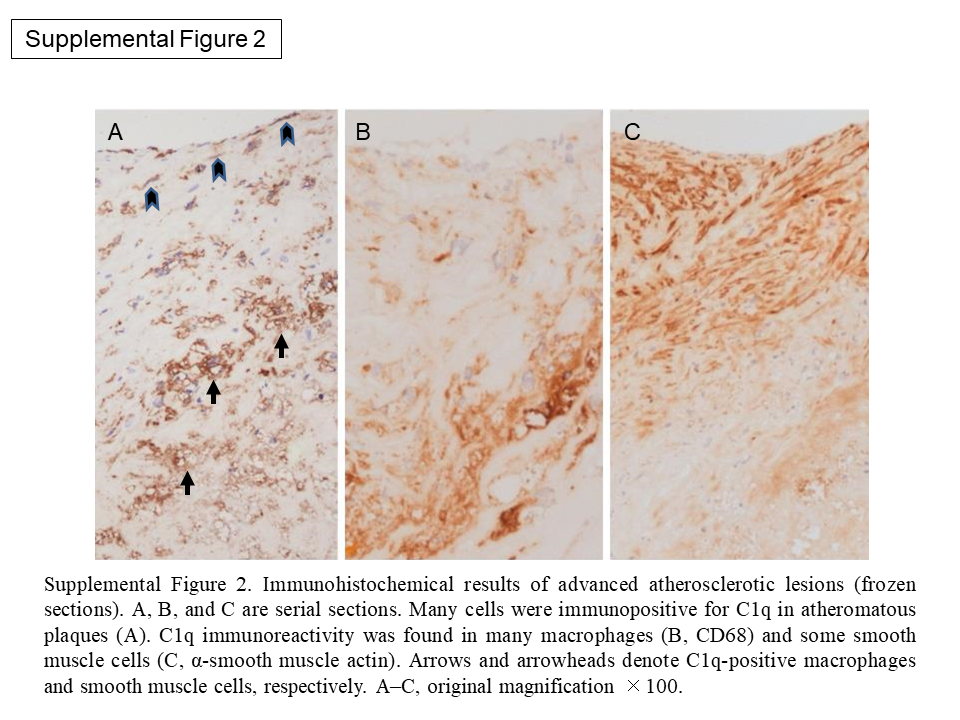

Supplement: S2 Fig — A, B and C are serial sections. Many cells are immunopositive for C1q in atheromatous plaque (A). C1q immunoreactivity is found in many macrophages (B, CD68) and some smooth muscle cells (C, α-smooth muscle actin). Arrows and arrowheads show C1q-positive macrophages and smooth muscle cell, respectively. A-C, original magnification x100. (TIF) [file pone.0262413.s002.TIF]

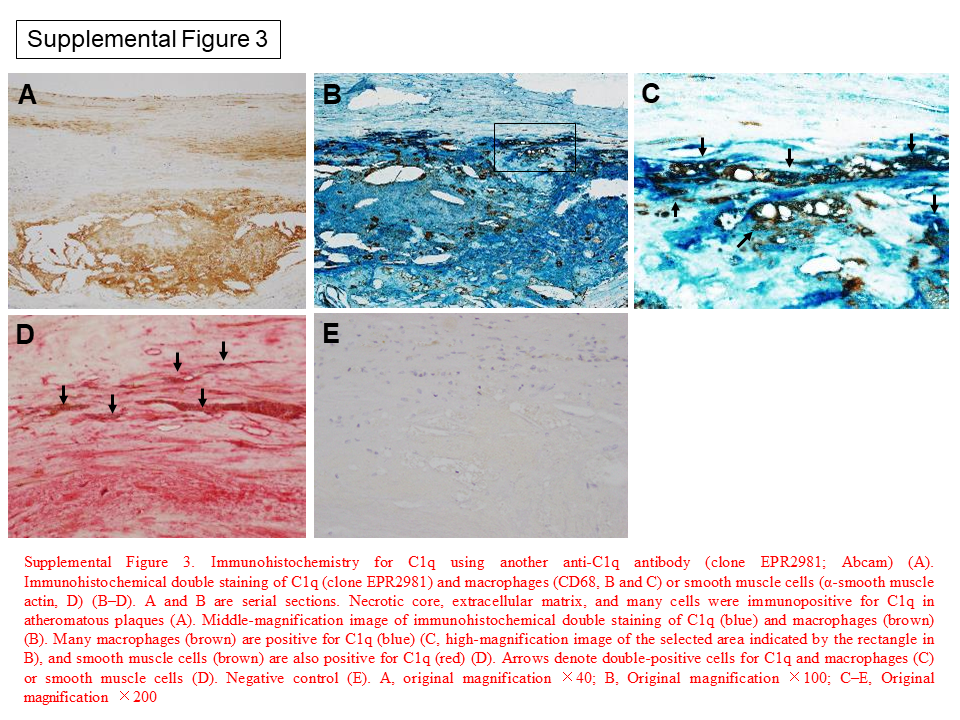

Supplement: S3 Fig — Immunohistochemistry for C1q using another anti-C1q antibody (clone EPR2981) (A). Immunohistochemical double staining of C1q (clone EPR2981) and macrophages (CD68, B and C) or smooth muscle cells (α-smooth muscle actin, D) (B–D). A and B are serial sections. Necrotic core, extracellular matrix, and many cells were immunopositive for C1q in atheromatous plaques (A). Middle-magnification image of immunohistochemical double staining of C1q (blue) and macrophages (brown) (B). Many macrophages (brown) are positive for C1q (blue) (C, high-magnification image of the selected area indicated by the rectangle in B), and smooth muscle cells (brown) are also positive for C1q (red) (D). Arrows denote double-positive cells for C1q and macrophages (C) or smooth muscle cells (D). Negative control (E). A, original magnification ×40; B, Original magnification ×100; C–E, Original magnification ×200. (TIF) [file pone.0262413.s003.TIF]

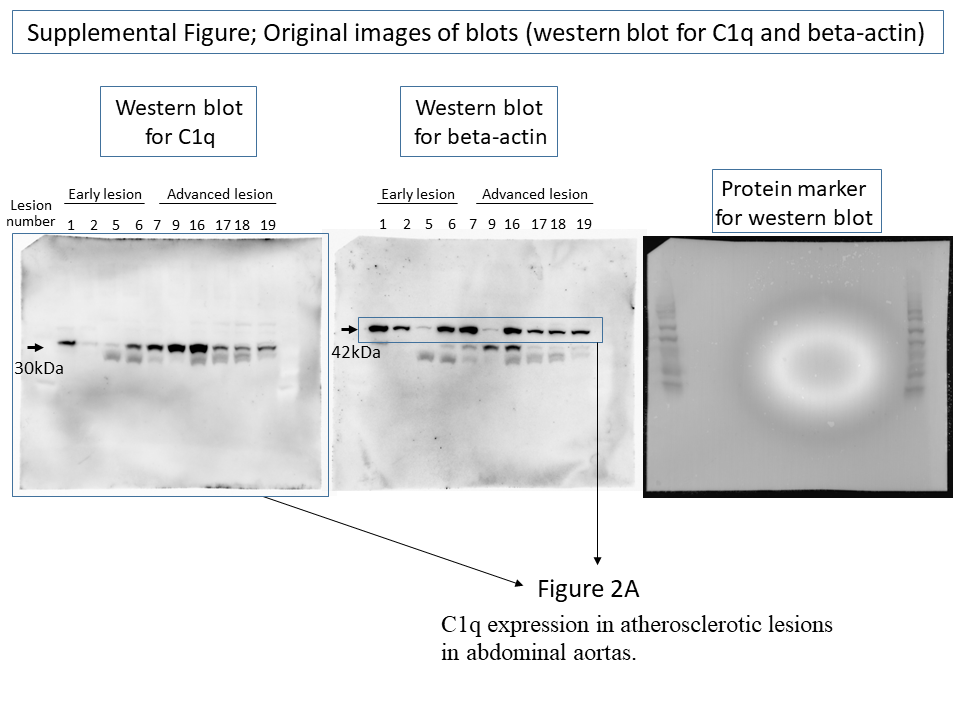

Supplement: S1 Raw images — (TIF) [file pone.0262413.s005.TIF]
