# Supplementary material for: Involvement of enhanced expression of classical complement C1q in atherosclerosis progression and plaque instability: C1q as an indicator of clinical outcome
Source: PLoS One. 2022 Jan 27;17(1):e0262413. doi: 10.1371/journal.pone.0262413 (PMC8794146; doi:10.1371/journal.pone.0262413)
Supplement: S1 Table — (DOCX) [file pone.0262413.s004.docx]

| Lesion number | Case number | Stain | Frozen section | Method | Lesion type |
| --- | --- | --- | --- | --- | --- |
| 1 | 1 | HE, IHC | 〇 | Western | AHA type II |
| 2 | 1 | HE, IHC |  | Western | AHA type II |
| 3 | 1 | HE, IHC |  | PCR | AHA type II |
| 4 | 1 | HE, IHC |  | PCR | AHA type II |
| 5 | 2 | HE, IHC | 〇 | Western | AHA type II |
| 6 | 2 | HE, IHC |  | Western | AHA type II |
| 7 | 3 | HE, IHC | 〇 | Western | AHA type VI |
| 8 | 3 | HE, IHC |  | PCR | AHA type VI |
| 9 | 4 | HE, IHC | 〇 | Western | AHA type VI |
| 10 | 4 | HE, IHC |  | PCR | AHA type V |
| 11 | 5 | HE, IHC |  | PCR | AHA type II |
| 12 | 5 | HE, IHC |  | PCR | AHA type II |
| 13 | 5 | HE, IHC |  | PCR | AHA type II |
| 14 | 6 | HE, IHC |  | PCR | AHA type VI |
| 15 | 6 | HE, IHC |  | PCR | AHA type VI |
| 16 | 7 | HE, IHC |  | Western | AHA type VI |
| 17 | 7 | HE, IHC |  | Western | AHA type VI |
| 18 | 8 | HE, IHC |  | Western | AHA type V |
| 19 | 8 | HE, IHC |  | Western | AHA type V |
| IHC, immunohistochemistry | | |  |  |  |
| Western, western blotting | | |  |  |  |
| PCR, real time-polymerase chain reaction | | | |  |  |
| AHA, American Heart Association | | | |  |  |
